# Navigating predictions at nanoscale: a comprehensive study of regression models in magnetic nanoparticle synthesis†

**DOI:** 10.1039/d4tb02052a

**Published:** 2024-11-04

**Authors:** Lukas Glänzer, Lennart Göpfert, Thomas Schmitz-Rode, Ioana Slabu

**Affiliations:** a Institute of Applied Medical Engineering, Helmholtz Institute, Medical Faculty, RWTH Aachen University Germany slabu@me.rwth-aachen.de +49 241 8089102

## Abstract

The applicability of magnetic nanoparticles (MNP) highly depends on their physical properties, especially their size. Synthesizing MNP with a specific size is challenging due to the large number of interdepend parameters during the synthesis that control their properties. In general, synthesis control cannot be described by white box approaches (empirical, simulation or physics based). To handle synthesis control, this study presents machine learning based approaches for predicting the size of MNP during their synthesis. A dataset comprising 17 synthesis parameters and the corresponding MNP sizes were analyzed. Eight regression algorithms (ridge, lasso, elastic net, decision trees, random forest, gradient boosting, support vectors and multilayer perceptron) were evaluated. The model performance was assessed *via* root mean squared error (RMSE), mean absolute error (MAE), mean absolute percentage error (MAPE) and standard deviation of residuals. Support vector regression (SVR) exhibited the lowest RMSE values of 3.44 and a standard deviation for the residuals of 5.13. SVR demonstrated a favorable balance between accuracy and consistency among these methods. Qualitative factors like adaptability to online learning and robustness against outliers were additionally considered. Altogether, SVR emerged as the most suitable approach to predict MNP sizes due to its ability to continuously learn from new data and resilience to noise, making it well-suited for real-time applications with varying data quality. In this way, a feasible optimization framework for automated and self-regulated MNP synthesis was implemented. Key challenges included the limited dataset size, potential violations of modeling assumptions, and sensitivity to hyperparameters. Strategies like data regularization, correlation analysis, and grid search for model hyperparameters were employed to mitigate these issues.

## Abstract 2

The transformative power of support vector regression in optimizing magnetic nanoparticle synthesis intricate relationships between process parameters and particle size, enabling the production of particles with tailored properties.

## Graphical abstract

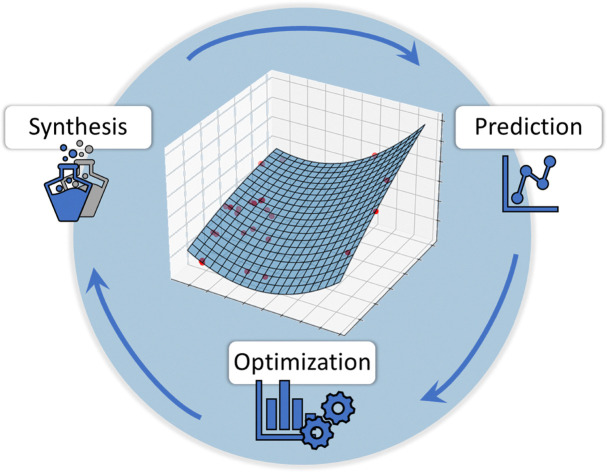

## 1. Introduction

Magnetic nanoparticles (MNP) are of high interest due to their unique magnetic properties and wide-ranging applications in various research fields, including biomedicine,^1–5^ environmental remediation,^6^ and catalysis.^7^ In the medical field, MNP have gained special attention in pharmacology, molecular imaging, and bio-sensing.^8,9^ Most promising future potential therapeutic applications of MNP include their use in localized magnetic hyperthermia cancer treatment and controlled drug delivery.^10–12^

Tailoring the size, narrowing size distribution, and achieving high phase purity of MNP can significantly impact their magnetic behaviour and performance for the different applications mentioned above.^13,14^ The ability to tailor the properties of MNP during synthesis has been highlighted as essential for optimizing their performance in specific applications like serving as contrast agents in magnetic resonance imaging (MRI), as tracers in magnetic particle imaging (MPI) or for magnetic hyperthermia for heating.^15,16^ However, the synthesis process involves numerous variables that can influence the resulting properties, making it challenging to predict and control the desired outcomes.^17–20^ Therefore, developing a robust predictive model that can accurately map the synthesis parameters to the target properties of MNP is of paramount importance. Such a model would enable researchers and manufacturers to design and produce MNP with custom-tailored properties, thereby enhancing their efficacy and expanding their potential applications.

Despite the significant advancements in MNP synthesis techniques, the underlying chemistry governing the formation and properties of these MNP is highly complex and not fully understood.^9,18,21,22^ The synthesis process involves intricate interactions between various parameters, such as precursor concentrations, reaction temperature or pH, which can have nonlinear and interdependent effects on the resulting MNP properties. This complexity makes it challenging to develop accurate theoretical models or derive closed-form equations that can reliably predict the desired properties based on the synthesis conditions.^23–25^ Machine learning algorithms, with their ability to learn from data and capture intricate patterns, offer a powerful approach to address these challenges. It is important to note that the data used in this study is extracted from a specific synthesis setup, with parameters that are unique to particular experimental configuration. Consequently, the data cannot be combined with information gathered from other setups, as similar parameters may not always be directly comparable between different synthesis systems. This specificity underscores the need for careful consideration when applying machine learning models across different experimental setups in the field of MNP synthesis. By training machine learning regression models on experimental synthesis data, this study aims to unravel the underlying relations between synthesis parameters and target properties, enabling accurate predictions and facilitating the design and production of MNP with custom-tailored characteristics specific to the underlying experimental setup. However, the presented framework is versatile and can be used for all sorts of manufacturing processes.

While the potential benefits of a predictive model are significant, several challenges must be addressed. Firstly, the available synthesis data is often limited, which can hinder the model's ability to generalize and make accurate predictions.^26–28^ Secondly, the synthesis process involves numerous variables, some of which may have nonlinear and interactive effects on the target properties, making the modelling task more complex.^29–32^

**Fig. 1:**
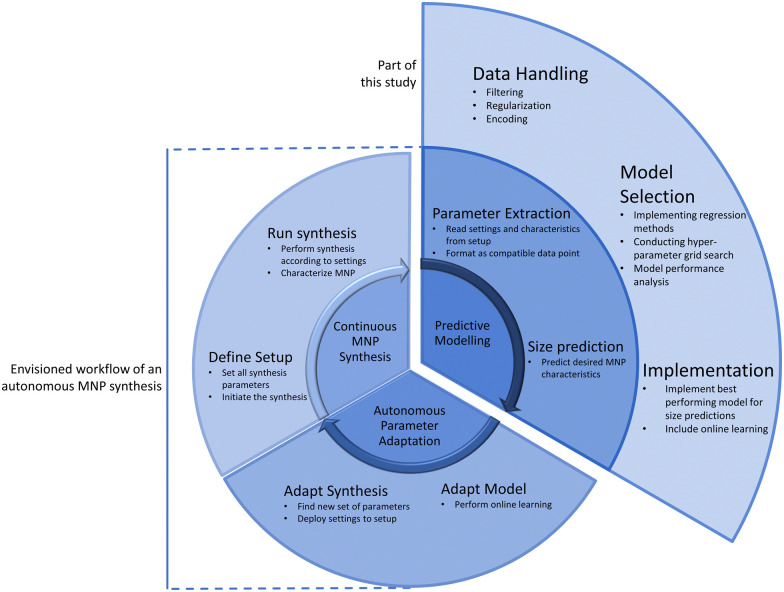
The envisioned workflow of an autonomous MNP synthesis process with machine learning optimization for the production target. This study aims to build the predictive modelling unit of this workflow. The necessary steps are given in the outer extension of the “Predictive Modelling” section.

Fig. 1 shows the envisioned workflow of an automated, self-regulated and targeted MNP production setting. A setup for a continuous MNP synthesis^33^ yields MNP within a narrow size range. However, an optimization of the process is needed to ensure MNP production with a targeted size. Here, a predictive model will be developed using the parameters of the synthesis setup described in ref. 33 as an input. The system parameters of the system will serve as training features whereas the MNP size will be the training target. The model should be able to output a new set of parameters for the synthesis setup to optimize production towards specific MNP sizes. With a regression model capable of performing online learning, the model can progressively adapt with the ongoing continuous synthesis and guide the production towards a defined target. Further, the predictive model should be capable of effectively capturing the complex relations between synthesis parameters and target properties of MNP. This model will then be the basis for the self-regulated parameter adaptation of the automated synthesis.

## 2. Material and methods

### 2.1 Data acquisition

The dataset encompasses 17 system parameters (*cf.* Section 2.2.1) extracted from a previously published synthesis setup,^33^ offering a detailed exploration of the MNP production process.

MNP synthesis involves two main stages: upstream and downstream processing. For this study, all data is extracted from the upstream process, as this is where the actual formation and size determination of the MNP occur. This crucial stage involves carefully controlling reaction conditions such as temperature, pH, and educt concentrations to nucleate and grow MNP with desired sizes and properties (*cf.*Table 1). Following the upstream synthesis, downstream processing involves purification steps to isolate the MNP and remove any unreacted educts or byproducts, as well as characterization techniques to verify the quality and attributes of the synthesized MNP. Here, the final MNP size is determined and added to the extracted data.

**Table 1:** The following table gives an overview of all parameters of the upstream synthesis (see Section 2.1) and further distinguishes between material parameters – parameters that are defined by the type of iron salts, bases or coating material – and synthesis settings – parameters that are settable before or during the running synthesis

Parameter type	Parameter group	Parameter name	Description
Material parameters	Iron salts	FeSO_4_·7H_2_O	Concentration in mol
		FeCl_3_·6H_2_O	Concentration in mol
		Iron ion ratio	Ratio of Fe^3+^/Fe^2+^
		V-IronSalts	Total educt volume in mL
	Base	NH_3_	Molarity in mol L^−1^
		pH	pH value of the base
		V-Base	Base volume in mL
	Coating	Coating type	Type of coating material
		V-Coating	Volume of coating material in mL
System settings	Temperature^a^	T-IronSalts	Temperature of the iron salts
		T-Base	Temperature of the base
		T-Coil	Temperature during crystallization
	Flowrate	FR-IronSalts	Flowrate of the iron salts in mL s^−1^
		FR-Base	Flowrate of the base in mL s^−1^
		FR-Coating	Flowrate of the coating material in mL s^−1^
	Reactor	Coil length	Length of the reaction coil in mm
		Quenching position	Quenching position in the coil in mm
Target	—	MNP size	Measured MNP size in nm

a The parameters include three temperatures for each of the iron salts, the base and the coil. Otherwise, temperature is set to “RT” (= room temperature). These parameters are included to ensure compatibility with future data where the temperatures might be set differently.

This study focuses on the prediction of the size of the synthesized MNP. Across the complete and unfiltered dataset, the MNP size exhibits a range from 4.0 to 90.21 nm with a median of 14.3 nm. While the range is constrained, this limitation facilitates an initial comparative study, even with a modest dataset size of 113 data points. The curated dataset provides a focused yet insightful examination of the interplay between system parameters and MNP size.

Although the dataset is limited in number of data points, adding data from other sources is infeasible. The data extracted from this synthesis is highly specific to the setup design. For example, the flow rate of an educt or the position of the quenching in the reactor is not directly linked to other synthesis setups. Thus, mixing information from multiple setups would yield wrong results.

### 2.2 Data preparation

#### 2.2.1 Data structure

The dataset captures a comprehensive set of parameters crucial for the synthesis of MNP. The parameters can be categorized into educt, base, temperature, flow rates, coating, reactor, and the resulting MNP size. Table 1 groups all parameters that were taken from the synthesis setup. The complete dataset is given in Table S1 (ESI†).

#### 2.2.2 Data filtering

Usually experimental data is documented laboratory environment and contains information which is not useful for the model development., *e.g.* dates and times, storage notes *etc.* Therefore, data filtering is necessary to ensure conciseness of the used data.

After a manual selection of potentially relevant features, an automated data filtering process was conducted with a specific focus on ensuring consistency within the dataset derived from the synthesis process. Accordingly, the dataset should correspond to a specific coating type, *i.e.*, citric acid. Filtering out data points with other coating types reduced the dataset to 101 data points. This strategic filtering approach aimed to emphasize these predominant coating type, which constituted most data points in the dataset. The rationale behind this filtering strategy was to enhance the relevance and alignment of data points, facilitating more meaningful results for size prediction without the influence on the coating type. Further, data points where the target variable was not included due to an erroneous synthesis were removed. Consequently, the dataset was reduced to 71 data points. This selective data filtering ensured that the dataset maintained a comparable structure, allowing for a more focused and meaningful analysis of the synthesis process and the resulting MNP sizes. Additionally, columns exhibiting constant values across all data points were excluded to maintain dataset conciseness (*e.g.* all temperatures were given as “RT”, see Table 1). The resulting dataset, now homogenized in terms of coating types, serve as the foundation for subsequent stages of data preparation and regression modelling.

#### 2.2.3 Outlier removal

Outlier removal prior to the model training is crucial, as these anomalous data points can significantly impact the model's performance and generalization ability. In the context of MNP synthesis, outliers may arise from various sources such as experimental errors or equipment malfunctions during data collection, temporary process disturbances or unusual operating conditions, sensor noise or calibration issues or human errors during data recording or sample handling.

The outlier removal process was refined by employing the median absolute deviation (MAD) principle.^34^ This method involves calculating the MAD value, a robust measure of data dispersion, and determining outliers based on their *z*-scores.^35^ Specifically, each data point with a *z*-score exceeding the median of *z*-scores plus 3 times the MAD value was identified as an outlier. This approach provides a more robust and resistant measure of dispersion, particularly suitable for datasets with potential skewness or non-normal distributions. Following this criterion, the dataset was pruned to 67 data points, underscoring a refined dataset less influenced by outliers.

The utilization of the MAD principle for outlier detection enhances the sensitivity of the process to variations in the dataset, ensuring a more reliable and statistically grounded approach to maintaining dataset integrity.

#### 2.2.4 Handling missing values

The treatment of missing values within the dataset involved a systematic approach to ensure the integrity and completeness of the data. The strategy employed was to fill missing values based on the nature of the data represented in each column. For columns representing the amount of an educt used in the synthesis, missing values were replaced with zeros, reflecting the absence of this specific educt in the synthesis. For other columns containing missing values, the approach involved replacing these gaps with the mean value of the respective column. This imputation strategy aimed to preserve the overall statistical characteristics of the dataset while ensuring that missing values did not compromise the quality of the analysis.

This standardized handling of missing values contributes to a consistent and complete dataset, ready for further analysis and regression modeling.

#### 2.2.5 Data normalization and encoding

A critical step in the data preparation process involves normalization and encoding to ensure the consistency and compatibility of the data for regression modeling. To standardize the scale of numeric features, the StandardScaler of the Python package scikit-learn^36^ was applied. This normalization technique transforms the data, ensuring that each feature has a mean of 0 and a standard deviation of 1. Normalization is imperative for regression models, particularly when dealing with features of varying magnitudes, as it aids in preventing certain features from disproportionately influencing the model.

Categorical variables, such as coating types, were subjected to one-hot encoding, again with the scikit-learn package. This method converts categorical variables into binary vectors, enabling the incorporation of categorical information into the regression models.

This dual approach of normalization and encoding ensures that the dataset is suitably prepared for regression modeling, allowing for an effective and unbiased exploration of the relations between features and the target variable, MNP size.

#### 2.2.6 Feature and target definition

Before the actual training, the features and target variables for the regression models are defined. The features comprise parameters associated with the MNP synthesis process, while the target variable represents the MNP size, which is the focal point of the predictive modeling. The feature set includes material parameters such educt concentrations and system settings such as temperatures, flowrates *etc.* (*cf.*Table 1). These features were carefully selected during the data preparation stage to ensure that they encapsulate critical information influencing the synthesis outcome. The target variable, MNP size, is the parameter to be predicted by the regression models. This essential variable guides the training and evaluation of the models, aligning with the primary objective of understanding the relations between synthesis parameters and resultant MNP size.

### 2.3 Regression models

#### 2.3.1 Model selection criteria

In the process of selecting regression models for this comparative study, specific criteria were established to ensure the appropriateness of each model for the dataset's unique characteristics and the research objectives. The chosen regression models were evaluated based on their capacity to handle a limited dataset effectively. Given the small sample size of 67 data points, models capable of generalizing well from limited data were prioritized to ensure robust performance and reliable predictions. Furthermore, as the dataset contains a high number of both interdependent and independent features, the prediction model is expected to efficiently handle this high-dimensional feature space. Precision in predicting MNP size was identified as a critical criterion. Models were assessed for their ability to provide accurate and precise predictions, minimizing errors in estimating the target variable. Additionally, considering the dynamic nature of MNP synthesis processes, models with online learning capabilities are preferred as they allow further model refinement. This enables the models to adapt and update their predictions with new data, ensuring continuous improvement and relevance of the model over time.

#### 2.3.2 Brief descriptions of tested methods

In this study, eight regression models were employed to predict MNP size, and their hyperparameters were meticulously optimized through an extensive grid search. The selected models and their detailed explanations are as follows:

Ridge regression is a linear regression variant that introduces regularization through a penalty term, known as the L2 penalty, which is added to the cost function.^37^ The regularization helps mitigate multicollinearity among the features, promoting stability and preventing overfitting. Ridge regression is particularly useful when dealing with datasets where many features are correlated.^38^

Like ridge, lasso regression incorporates regularization but uses the absolute values of coefficients, implementing the L1 penalty. This has the effect of inducing sparsity in the model, leading to automatic feature selection.^32^ Lasso is advantageous when dealing with high-dimensional datasets, effectively narrowing down to the most influential predictors.^39^

Elastic net regression combines the attributes of ridge and lasso and employs both the L1 and L2 penalties. This hybrid approach offers a balanced regularization that handles correlated features effectively while still promoting sparsity. Elastic net is suitable for datasets with a mix of correlated and independent features.^40^

Decision tree regression constructs a tree-like model by recursively partitioning the dataset based on feature conditions where each node in the tree represents a decision.^41^ The final prediction is made by aggregating these decisions. Decision trees are adept at capturing non-linear relations and are resilient to outliers, making them suitable for diverse datasets.^42^

Random forest regression build an extension of decision trees. They aggregate predictions from multiple trees, creating an ensemble.^43^ This ensemble learning technique improves predictive accuracy and reduces overfitting. Random forest is robust, handles high-dimensional data well, and provides feature importance rankings.^44^

Gradient boosting regression is another powerful ensemble learning technique. It builds an additive model of weak learners, typically shallow decision trees.^45^ It sequentially corrects errors in predictions, capturing intricate patterns and relations in the data. Gradient boosting excels in capturing complex relations and is less prone to overfitting.^46^

Support vector regression predicts continuous outcomes by mapping data points into a high-dimensional space using a kernel function.^47,48^ It is particularly effective in capturing non-linear relations in high-dimensional spaces. This approach is advantageous when dealing with datasets with complex decision boundaries and sparse feature spaces.^49^

Multilayer perceptron regression utilized an artificial neural network with multiple layers for its predictions. It can capture intricate patterns in data through a complex network of interconnected nodes. This regression approach is suitable for modeling complex relations but may require careful tuning to avoid overfitting.^50^

The subsequent grid search over hyperparameters aimed to fine-tune each model's performance, ensuring their effectiveness in predicting MNP size.

### 2.4 Cross-validation

To assess the generalization performance of the regression models and ensure their robustness, cross-validation techniques were employed. Cross-validation involves partitioning the dataset into subsets for training and testing, iteratively rotating through different combinations to evaluate the model's performance comprehensively. In this study, leave-one-out cross-validation (LOOCV) was specifically chosen. LOOCV involves using a single data point as the validation set while the remaining data points constitute the training set. This process is repeated for each data point, providing an exhaustive assessment of the model's ability to generalize.

The selection of LOOCV aligns with the limited size of the dataset, maximizing the use of available data for both training and validation.^51^ This approach minimizes bias introduced by the small sample size^52^ and provides a reliable estimate of the models’ performance under different conditions as well as offering insights into their ability to generalize to new data points.

In the context of cross-validation, it is important to note that the primary purpose is to determine the optimal hyperparameters for each model. The process involves assessing model performance across various folds to find the configuration that generalizes well to unseen data. Following hyperparameter tuning, the final training is conducted using the identified optimal settings, combined with a random 80–20 split of the dataset. This approach ensures a robust evaluation of the models and enhances their adaptability to new data points beyond the seen training set.

### 2.5 Statistical analysis

To comprehensively evaluate the performance and variability of the regression models in predicting MNP size, descriptive statistics including the root mean squared error (RMSE), mean absolute error (MAE), mean absolute percentage error (MAPE) and the standard deviation of residuals were computed.

RMSE is a commonly used metric for assessing the accuracy of regression models, providing a measure of the average magnitude of the prediction errors. Mathematically, RMSE is calculated as the square root of the mean of the squared differences between predicted and actual values. The formula is given by:
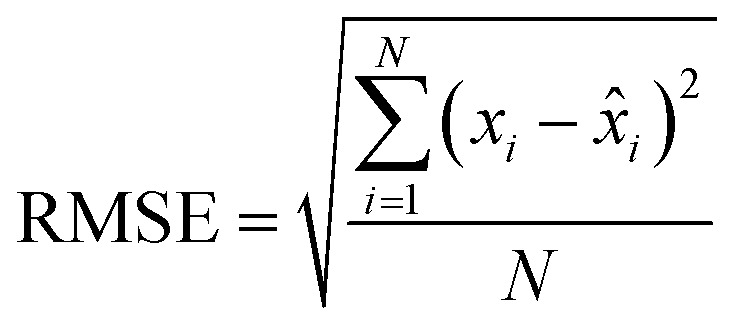
where *x*_*i*_ represents the actual values, *x̂*_*i*_ represents the predicted values, and *N* is the number of data points.

RMSE is particularly suitable for this study due to its sensitivity to large errors. By squaring the differences between predicted and actual values, RMSE penalizes larger errors more heavily, providing a balanced representation of the overall model performance. Given the nature of predicting MNP size, where precision in the predictions is crucial, RMSE serves as a reliable metric for assessing the accuracy of regression models trained on the synthesized datasets.

Similarly, MAE is also a common metric for assessing the accuracy of regression models. In contrast to RMSE, MAE computes the average of the absolute prediction errors, thus putting less emphasis on large errors. Therefore, it is sensible to compare models on both metrics to gain additional information on the models’ handling of extreme values. MAE's formula is given by:
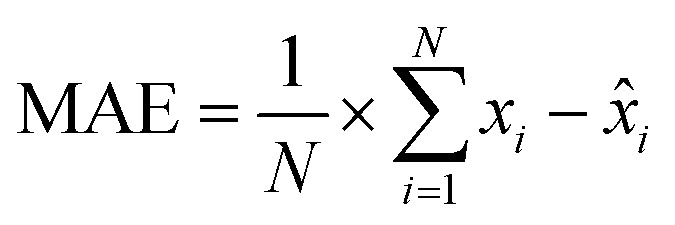
with *x*_*i*_, *x̂*_*i*_ and *N* as before.

MAPE is a widely used metric for evaluating the accuracy of prediction models. It expresses the average absolute error as a percentage of the actual values, making it an intuitive and scale-independent measure. This characteristic allows for easy comparison of model performance across different datasets or time series, regardless of their magnitudes. It helps in assessing how far off predictions are on average, with lower MAPE values indicating more accurate predictions. Its formula is given by:
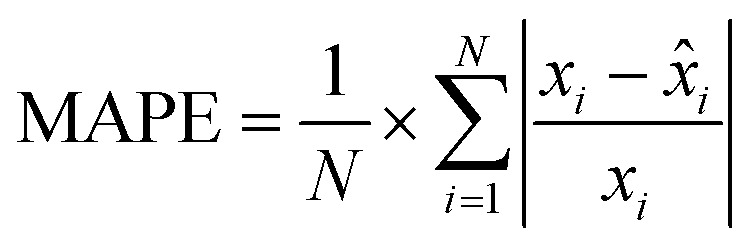
with *x*_*i*_, *x̂*_*i*_ and *N* as before.

The standard deviation of residuals quantifies the spread or variability of prediction errors around the regression line. A lower standard deviation implies more consistent model predictions, while higher values may suggest variability in prediction errors, known as heteroscedasticity.

### 2.6 Used software and hardware

The implementation of regression models and the analysis of data were conducted using Python as the primary programming language. Key libraries and tools employed include scikit-learn^36^ for regression modeling and matplotlib^53^ for data visualizations.

## 3. Results

This chapter presents the outcomes of the conducted MNP synthesis regressions. Each tested regression method is defined by its determined hyperparameters shown in Table 2. For a better visualization and interpretation of the dataset, descriptive statistics were used, outlining the distribution and central tendencies of MNP sizes. A correlation analysis reveals relations between individual system parameters and MNP sizes, shedding light on influencing factors in the synthesis process. The qualitative and quantitative evaluation of different regression models assesses their predictive performance and suitability for MNP size prediction (*cf.* Sections 3.3 and 3.4).

**Table 2:** Regression models and the respective hyperparameters and tested ranges. All numerical ranges are denoted as intervals enclosing both endpoints. For intervals with real numbers, 250 values evenly spread over the interval were tested. The hyperparameters are named according to the models’ implementation in the scikit-learn libraries

Model	Hyperparameter	Tested range/values	Best value
Ridge	alpha	[0.0, 25.0]	20.2
Lasso	alpha	[0.0, 2.5]	0.26
	tol	[0.0, 0.25]	0.098
Elastic net	alpha	[0.0, 1.0]	0.532
	L1_ratio	[0.01, 1.0]	0.01
Decision tree	max_depth	[1, 10]	3
	min_samples_split	[2, 10]	2
	min_samples_leaf	[1, 5]	1
Random forest	n_estimators	[100, 300]	117
	max_depth	[1, 10]	5
	min_samples_split	[2, 10]	3
	min_samples_leaf	[1, 5]	1
Gradient boosting	n_estimators	[100, 300]	275
	max_depth	[1, 10]	6
	min_samples_split	[2, 10]	2
	learning_rate	[0.0001, 0.1]	0.009
Support vector	C	[100.0, 200.0]	154.17
	gamma	[0.0, 1.0]	0.71
	epsilon	[0.0, 1.0]	0.1
	kernel	[linear, poly, rbf, sigmoid]	rbf
Gradient boosting	n_estimators	[100, 300]	275
	max_depth	[1, 10]	6
	min_samples_split	[2, 10]	2
	learning_rate	[0.0001, 0.1]	0.009
Multilayer perceptron	activation	[logistic, tanh, relu, identity]	relu
	solver	[adam, sgd, lbfgs]	sgd
	alpha	[0.0, 3.0]	1.1
	learning_rate	[constant, invscaling, adaptive]	adaptive

Additionally, a sensitivity analysis underscores the relative impact of system parameters on the variability in predicted MNP sizes.

### 3.1 Hyperparameter study

Table 2 shows each model, its tuned hyperparameters and their tested ranges. All methods were implemented in Python by utilizing scikit-learn. The hyperparameters are thus named according to the implementation. To capture the complexity of hyperparameter tuning, sensible ranges must be chosen for testing the hyperparameters. For categorical hyperparameters (*e.g.* kernel, activation function, *etc.*) all available options were tested. For numerical parameters, multiple grid searches were performed, each varying the tested ranges for each hyperparameter. The resulting grid search is shown in Table 2 and displays large ranges for each parameter. Table 2 denotes the tested ranges as intervals with both endpoints enclosed. For hyperparameters with integer values (*e.g.* max_depth, min_samples_split, *etc.*), all values within the interval were tested. For hyperparameters with real values (*e.g.* alpha, gamma, epsilon, *etc.*), 250 values evenly spread over the interval were tested. For all tested hyperparameters, the identified best value lies well within the respectively tested interval, indicating suitable interval ranges.

### 3.2 Descriptive statistics

**Fig. 2:**
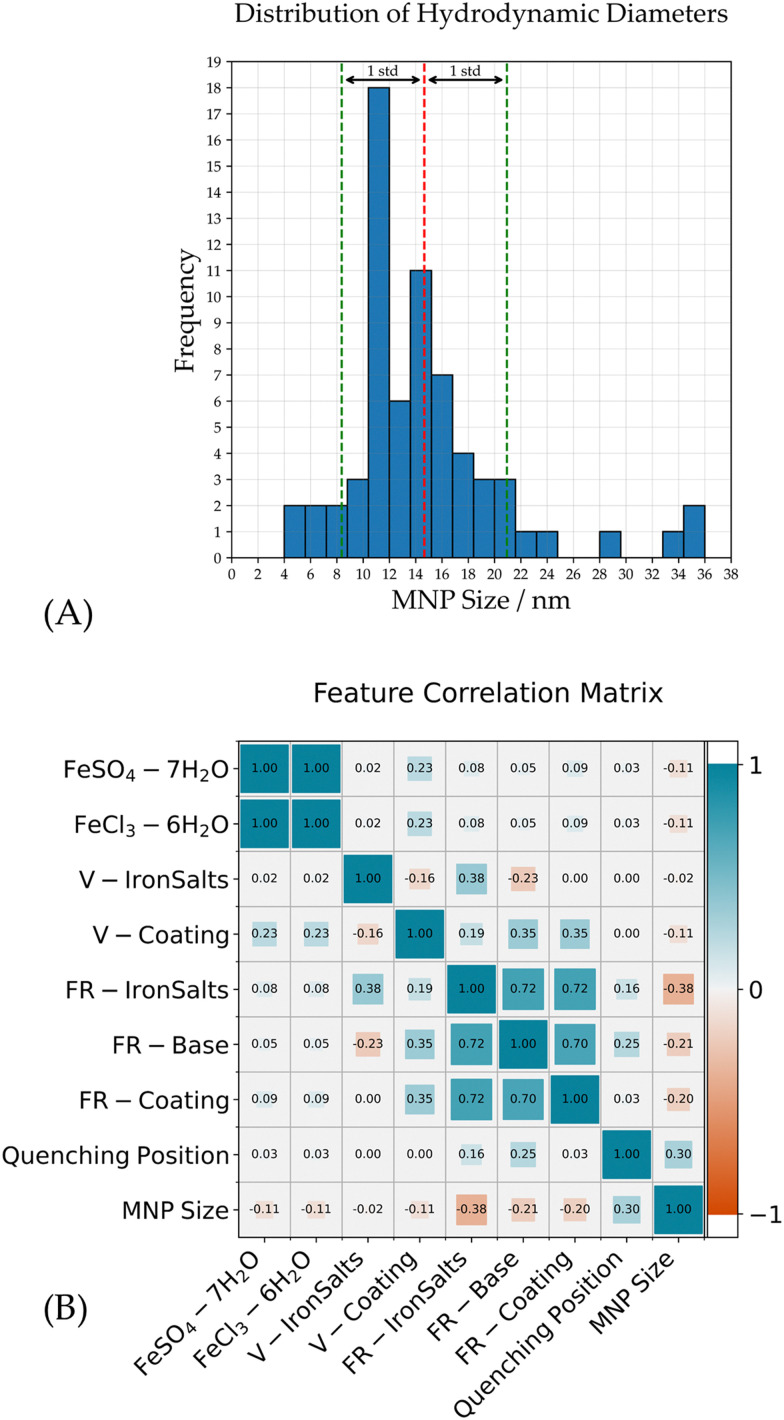
(A) A histogram of the MNP size distribution. The mean value is visualized by the red dashed line and the standard deviation by the green dashed lines. (B) The correlation matrix of all variables. Values are calculated by Pearson correlation coefficient. Blue indicates positive correlation; red indicates negative correlation. The magnitude of the correlations is indicated by color intensity and square size.

This section provides a brief overview of the distribution and central tendencies of the MNP sizes. After filtering and outlier removal (see Section 2.2), the dataset consisted of 67 data points. Within this data, the MNP sizes have a mean value of 14.65 nm, with a standard deviation of 6.3 nm, reflecting the narrow dispersion of values around the mean. The size range spans from a minimum of 4.0 nm to a maximum of 36.0 nm. Fig. 2(A) visualizes the MNP size distribution.

### 3.3 Correlation analysis

Fig. 2(B) shows the correlation matrix of the variables involved in the MNP synthesis process. The correlation value is calculated using the Pearson correlation coefficient. These variables, except for the MNP size, represent the parameters that are manually set at the start of the synthesis process.

The correlation matrix reveals several instances of moderate or strong correlations between individual variables. It is important to note that these correlations do not imply causation or influence between the parameters, as they are all manually set before the synthesis process. They merely reflect the relations in the chosen settings for these parameters. These relations are important to consider when choosing regression models, as potential multicollinearity influences regression performances. Noticeable correlations can be identified as follows:

• Perfect correlation: FeSO_4_·7H_2_O and FeCl_3_·6H_2_O have a perfect positive correlation of 1.0. This suggests that these two variables, which represent different iron salts used in the synthesis, might have similar effects on the MNP size.

• High positive correlation: all three flowrates have a high positive correlation of −0.7 or 0.72. This indicates that an increase in the flow rate is usually done simultaneously.

• Moderate positive correlation: V-ironSalts and FR-ironSalts have a positive correlation of 0.38. This indicates that the volume of the iron salt and the pressure of the iron salts are increased together in the synthesis settings. V-Coating, FR-base and FR-coating have a positive correlation of 0.35. This suggests that the flow rates and mass of the coating agent are increased together in the synthesis settings.

• Moderate negative correlation: V-ironSalts and FR-Base have a moderate negative correlation of −0.23. This suggests that an increase in the volume of the iron salt is accompanied by a decrease in the pressure of the ammonia water in the synthesis settings.

• Multicollinearity: the variables V-coating, FR-ironSalts, FR-base and FR-coating show correlations with each other. This suggests potential multicollinearity issues that may impact regression model reliability.

Looking at the correlations with the MNP size, no strong correlations can be identified:

• Moderate correlation: FR-ironSalts and the quenching position show moderate positive correlations with the MNP size of around −0.38 and 0.3, respectively.

• Little correlation: FR-base and FR-coating show little negative correlations with the MNP size with values around −0.2.

None of the variables shows a strong correlation (above 0.5 or below −0.5) with the MNP size. This suggests that no single variable has a significant impact on its own.

### 3.4 Regression model performance

**Fig. 3:**
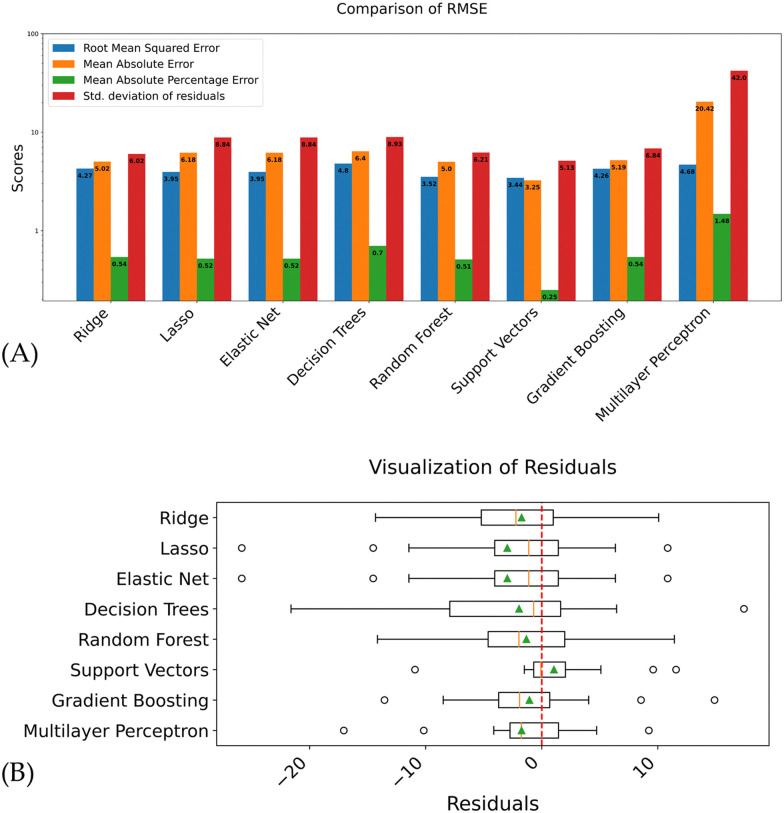
(A) Bar chart with prediction results of the trained models for the validation set. The blue bars represent the RMSE values, orange the MAE values, green the MAPE values and red the standard deviation of residuals. (B) Visualizes the distribution of the residuals of the validation set. The dashed line shows the ideal value of zero for residuals, the orange bars and triangles in the boxplots show the median and mean residual, respectively. Circles denote individual outliers.

This subsection aims to evaluate and compare the performance of various regression models in predicting the size of MNP based on the existing data. Four key metrics, the root mean square error (RMSE), the mean absolute error (MAE), the mean absolute percentage error (MAPE) and the standard deviation of residuals, are employed for this comparative analysis. The RMSE and MAE are measures of the average magnitude of the residuals or prediction errors. MAE is less sensitive to outliers than RMSE, whereas RMSE is more desirable when large errors should be avoided. MAPE is a measure of prediction accuracy that expresses the average absolute error as a percentage of the actual values, providing a scale-independent metric for comparison. The standard deviation of residuals provides an understanding of the spread of residuals around the mean residual, which ideally should be zero. Lower values of all of these metrics indicate better predictive performance. The models’ performances will be compared using bar charts, visually depicting the different scores (see Fig. 3(A)).

Additionally, box plots of residuals will be utilized to offer a detailed examination of the distribution of prediction errors across different models (see Fig. 3(B)).

Among the evaluated methods, the support vector regression model demonstrated superior performance across all metrics. It achieved the lowest RMSE (3.44), MAE (3.25), MAPE (0.25), and standard deviation of residuals (5.13), indicating high accuracy and consistency in its predictions. The random forest model emerged as the second-best performer, exhibiting low RMSE (3.52) and competitive MAE (5.0) and MAPE (0.51) values, suggesting a good balance between accuracy and consistency.

Interestingly, the lasso and elastic net models showed identical performance across all metrics, with moderate RMSE (3.95) but higher MAE (6.18) compared to the top performers. This similarity in results suggests that the L1 regularization component dominates in the elastic net model, effectively reducing it to a lasso model in this case. The ridge regression model demonstrated moderate performance across metrics, with a notably lower standard deviation of residuals (6.02) compared to lasso and elastic net, indicating more consistent predictions. Ensemble methods, particularly random forest and gradient boosting, generally performed well. However, the decision tree model underperformed compared to these ensemble methods, with higher RMSE (4.8) and MAPE (0.7) values indicating less accurate predictions. This underscores the advantages of ensemble techniques in improving model robustness and predictive accuracy. The multilayer perceptron model showed the poorest performance across all metrics, with extremely high MAE (20.42), MAPE (1.48), and standard deviation of residuals (42.0). These results suggest potential overfitting or an unsuitable architecture for the problem at hand, highlighting the importance of careful model selection and hyperparameter tuning in neural network applications.

Looking at the distribution of residuals, the median values for all models were close to zero with a little skewness in the negative direction, indicating that the models’ predictions are slightly biased on average. Ridge, lasso, elastic net and random forest all exhibited similar interquartile ranges (IQRs), suggesting comparable variability in the residuals. The decision tree regression model demonstrated a larger IQR, indicating more variability in the residuals. Support vectors, gradient boosting and multilayer perceptron show a smaller IQR, indicating less variability in the predictions. Models with a small range fulfill the requirement of precision. Also, the median residual of the support vectors is very close to zero, suggesting unbiased predictions on average. A median close to zero fulfills the requirement of accuracy.

### 3.5 Selection of the most suitable regression model

The evaluation of various regression models (see Fig. 3) reveals that random forests, and support vectors exhibit the best prediction accuracy. The support vectors demonstrate more centralized residuals compared to random forests, which exhibits a slight negative skewness, and a significantly larger range. Gradient boosting showed less prediction accuracy but had a much smaller range than random forests.

The support vectors demonstrated achieved a low MAPE value of 0.25, which is less than half that of the next best model, indicating its superior ability to generate predictions that closely align with actual values across the dataset.

In addition to quantitative measures, qualitative considerations are crucial for selecting the most suitable model. The integration of online learning in the MNP production necessitates a model capable of adaptation. While purely tree-based methods, *e.g.* decision trees and random forest, may face challenges in this regard, gradient boosting and support vectors offer promising capabilities. Both gradient boosting and support vectors excel in modeling complex nonlinear relations and handling correlated features. Gradient boosting's inherent tree structure enhances interpretability but complicates online learning adaptations, as each new chunk of data requires the addition of a new weak classifier, potentially leading to increased model complexity. Support vectors exhibits ease of adaptability to online learning, making it a favorable choice for real-time applications. Moreover, Gradient boosting's tendency to overfit on small datasets may result in longer learning times during the online learning phase compared to support vectors. Additionally, support vectors provides robustness against outliers and noise in the data, which is particularly beneficial in practice where data quality may vary. However, it is important to note that support vectors’ performance may be sensitive to the selection of hyperparameters, requiring careful tuning for optimal results. In contrast, gradient boosting tends to be more robust to hyperparameter settings, offering more straightforward implementation and potentially shorter development times.

**Fig. 4:**
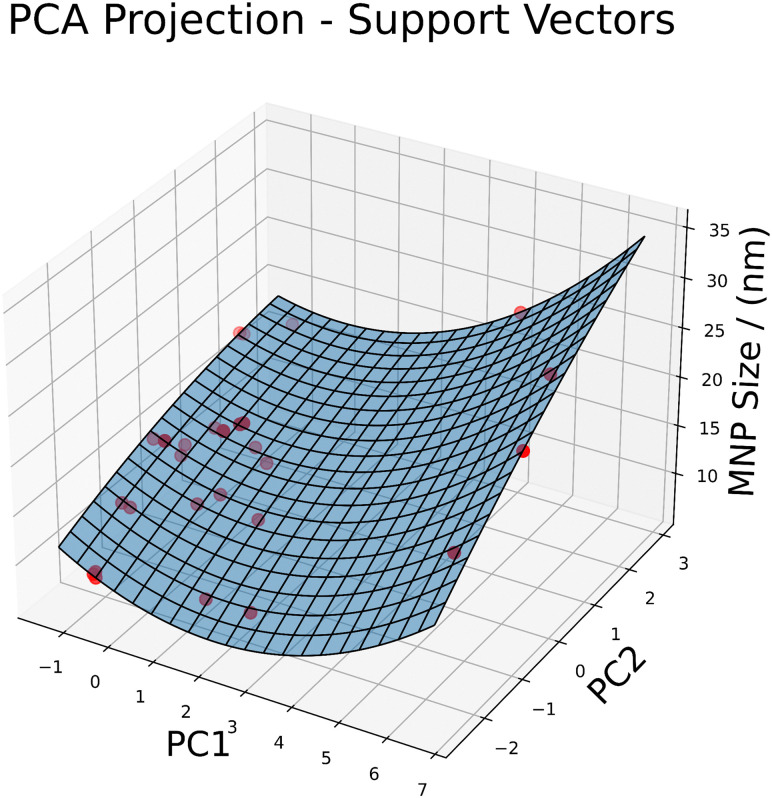
3D Surface plot of principal component analysis (PCA) applied to the support vector regression model. The original dataset, comprising 17 features, was transformed into a 3-dimensional space using PCA, with the first two principal components (PC1 and PC2) plotted on the *x* and *y* axes, respectively, and the SVR predictions on the *z* axis.

For the above-mentioned reasons, *i.e.* the achieved accuracy and precision as well as the ability to perform online learning, we selected the support vector regression as method of choice to be implemented in the overall MNP optimization framework. Fig. 4 shows the resulting prediction surface for the support vectors model. The shown surface is a projection from the 17-dimensional feature space to the 3-dimensional for better visualization. The projection was performed using principal component analysis (PCA). More PCA plots for each model from different angles are shown in S2 to show the different characteristics of each model and their predictions. PCA is a dimensionality reduction technique that transforms a high-dimensional dataset into a lower-dimensional space while preserving as much variance as possible. In this study, PCA was applied to reduce the original 17-dimensional feature space to two principal components. These components capture the most significant patterns in the data, allowing for a more manageable and interpretable visualization. Each principal component (PC) is a linear combination of original features. The coefficients – or loadings – of these combinations indicate how much each original feature contributes to the PC. Features with high absolute values in the loadings matrix are the ones that contribute most to the corresponding PC. The sign indicates the direction of the relations between the feature and the principal component.

The loadings shown in Table 3 show, that FeSO_4_·7H_2_O, FR-ironSalts and FR-coating for PC1 and FeCl_3_·6H_2_O for PC2 are strong contributors, meaning they have the strongest influence on the target variable, the MNP size.

**Table 3:** Loadings for each feature and each principal component. High absolute values indicate a strong contribution of the feature, the sign indicates whether the contribution is in positive or negative direction. Features with a “0.0” entry were rounded to the fourth decimal place thus showed non-zero but negligible contribution. Features with a “dash” entry showed actual zero contribution because they were constant

Parameter name	PCA loadings	
	Principal component 1 (PC1)	Principal component 2 (PC2)
FeSO_4_·7H_2_O	−0.5629	0.0987
FeCl_3_·6H_2_O	−0.1506	0.9122
Iron ion ratio	—	—
V-IronSalts	−0.284	−0.344
NH_3_	—	—
pH	—	—
V-Base	—	—
Coating type	—	—
V-Coating	0.0	0.0
T-IronSalts	—	—
T-Base	—	—
T-Coil	—	—
FR-IronSalts	−0.5432	−0.1982
FR-Base	0.0	0.0
FR-Coating	−0.5336	0.0233
Coil length	—	—
Quenching position	0.0	0.0

## 4. Discussion

This study aimed to find the most suitable machine learning regression predictor for the size of MNP during their synthesis. Various regression methods were evaluated based on performance metrics such as RMSE and standard deviation of residuals. Among the evaluated methods ridge, random forests, and support vector regression (SVR) demonstrated superior performance in predicting MNP sizes. They exhibited small RMSE and MAE values showing good prediction accuracy; but only SVR exhibited centralized residuals, indicating consistent prediction errors, while ridge and random forest showed a slight positive skewness and a large range. Additionally, SVR showed a significantly lower MAPE value of 0.25 than any other model. Qualitative considerations, such as adaptability to online learning and modelling of complex relations, favoured gradient boosting and SVR. Gradient boosting is expected to be suitable for complex non-linear data but could not outperform the other shown methods. Lastly, the PCA visualizations presented in Fig. 4 and Fig. S2 (ESI†) clearly show, that the resulting SVR model captures the training data best. All other models show significant deviations in their predictions from the surface plot. Thus, SVR emerged as the most suitable model due to its ease of adaptability to online learning, robustness against outliers and noise and suitability for real-time applications with varying data quality as well as its leading quantitative performance.

Machine learning applications offer numerous advantages for both predicting MNP sizes and gaining insights into the synthesis process.^54^ This study has shown that SVR can be a very promising and capable technique outperforming other methods. The used performance metrics such as root mean square error (RMSE), mean absolute error (MAE), mean absolute percentage error (MAPE) and the standard deviation of residuals clearly demonstrate this (*cf.*Fig. 3). These measures were chosen as they have proven to yield in general a comprehensive assessment of model performance.^55^ These metrics effectively capture the accuracy and consistency of predictions, enabling a thorough comparison of different regression methods.^56,57^

Moreover, the selected regression models, particularly SVR, exhibited robustness in handling complex nonlinear relations and correlated features. This capability is essential for accurately modelling the intricate dynamics of nanoparticle synthesis, where multiple parameters interact to influence the final product.^58^

SVR further distinguishes itself by its adaptability to online learning,^59–61^ making it well-suited for real-time applications and dynamic environments.^59^ This feature enables the model to continuously update and refine its predictions as new data becomes available, enhancing its practical utility in industrial settings. Prediction accuracy may vary or be biased due to a skewed dataset, but in the next steps we will incorporate online learning, as indicated in Fig. 1, to counteract these effects. A continuously increasing dataset will eventually become less biased or skewed. SVR's easy adaptation to incorporate online learning will leverage the incoming data from the synthesis setup and continuously learn the inherent process parameters of MNP synthesis and increase its prediction accuracy by proposing new parameters for the setup.

This continuous learning process was shown to lead promising results with the data used in this study, yet it requires rigorous validation to ensure the model's predictions remain accurate and reliable over time. Such validation involves comprehensive testing under various operating conditions, long-term stability assessments, and comparison with traditional optimization methods. Subsequent investigations will delve into the long-term performance of the online learning system, its adaptability to process drift, and its potential for autonomous optimization of MNP synthesis parameters.

The application of SVR for predicting magnetic MNP sizes presented several limitations and challenges, particularly due to dealing with small datasets.^26^ Small datasets inherently pose significant obstacles in machine learning endeavours, including issues related to data diversity, noise, and imbalance.^27,28^ This limited diversity can lead to SVR models that perform poorly when applied to new MNP systems outside the training data.^62,63^ To address data diversity, noise and imbalance, techniques such as data augmentation^64^ or online learning^65^ may be applied.

Data augmentation is a widely used and a well-established technique to handle data sparsity, however, since the synthesis process itself and thus its parameter settings are generally not well understood, the identification of such new settings is subject to this study. Regarding good practice for data augmentation,^29^ this ultimately makes applying data augmentation impossible for this study as no sensible new data points can be generated.

Online learning is a machine learning technique making the model capable of iteratively adding new data points from a pool of unlabelled or of newly generated data, and then incorporating these data into the training set to improve its performance.^66–68^ In the context of this study, online learning can be used to retrain the SVR model with the updated training set whenever new data are available, *i.e.* new syntheses were performed. However, because creating new data by synthesizing new MNP is time consuming the effectivity of online learning has to be subject of further studies. To handle these biases in the data and include newly available data, online learning will be implemented as a next step to continuously learn from the synthesis setup and thus increase the training data.

The hyperparameter settings of the conducted grid search for this study were extensively tested. Multiple grid searches were conducted to verify their results and carefully choose sensible hyperparameters. This is necessary, because SVR, as well as other machine learning methods, exhibits a high sensitivity to hyperparameter settings.^69,70^ The selection of appropriate kernel functions, regularization parameters, and other hyperparameters significantly influences the model's performance. Given the inherent challenges posed by small datasets, careful optimization of these parameters becomes paramount to prevent overfitting and ensure robust generalization to unseen data.^69,71^ Techniques such as grid search^72^ have been explored to enhance the performance of SVR models in such scenarios. For this study, grid search proved to be a sufficient and reliable method for hyperparameter tuning. Unlike random search or Bayesian optimization, grid search guarantees finding the best solution within the defined parameter ranges, providing a comprehensive and systematic exploration of the hyperparameter space. This exhaustive approach is especially valuable when dealing with multiple regression types, as it ensures that no potentially optimal configuration is overlooked. Furthermore, the computational costs associated with grid search were negligible, allowing for a thorough examination of the parameter space without compromising time or resources.

Moreover, the process of model training and validation presents its own set of challenges in the context of small datasets.^73,74^ Traditional validation methods, like cross-validation, may not be as effective with small datasets because splitting the data further reduces the amount available for training and validation, potentially leading to unreliable performance estimates.^75^ The employed leave-one-out-cross-validation (*cf.* Section 2.4) validates each data point against all others and thus tries to maximize the performance estimation reliability during the performed hyperparameter grid search.

Another measure to ensure robustness of the trained models, data regularization was implemented to prevent overfitting by penalizing complex models. As shown in Fig. 2(B), there are weak, but not negligible multicollinearities in the selected features of the dataset. It is imperative to acknowledge the assumptions underlying machine learning models regarding the independence of observations and the distribution of input features. Failure to meet these assumptions can significantly impact the model's learning capability and predictive accuracy.^29,30^ Strategies to avoid multicollinearity are a proper feature selection and data regularization.^31,32^

With this successful implementation, the study's methodology of using SVR to predict the outcome of MNP synthesis has broader applications. Similar supervised learning approaches could be applied to other manufacturing processes or material property testing, offering potential benefits for optimizing processes, predicting outcomes, and improving product quality across various industries. For example, in the field of pharmaceuticals, SVR models could be employed to predict the properties of drug formulations based on the synthesis parameters and raw material characteristics which could enable the optimization of drug manufacturing processes, leading to improved product quality and consistency.^76,77^ Machine learning regression is already employed in many applications for material synthesis and property prediction like constructing metal matrix composites, predicting molecular properties of new materials of nanomaterials.^77,78^

## 5. Conclusions

In conclusion, this study implemented and evaluated eight machine learning regression techniques to find a suitable method for MNP property prediction for a specific synthesis process. Support vector regression proved to be the most suitable method for a small, yet complex data set of 113 data points. It showed high accuracy of 3.44 nm and robust predictions with narrow distributed errors. Further, it features an easy incorporation of online learning techniques for improvement of the prediction accuracy. Within the synthesis framework the feasibility of optimizing MNP sizes using support vector regression was demonstrated. By leveraging the existing synthesis data, a model was trained to predict the desired process parameters, enabling the production of MNP with custom-tailored sizes. The successful implementation of such a model streamlines the synthesis process, reduce experimental efforts, and accelerate the development of advanced MNP-based technologies. This provides a major step towards fully automated and self-regulated MNP synthesis and beyond.

## Author contributions

Conceptualization, L. Glänzer and I. Slabu; methodology, L. Glänzer; software, L. Glänzer; validation, L. Glänzer and I. Slabu; formal analysis, L. Glänzer; investigation, L. Glänzer; resources, I. Slabu and T. Schmitz-Rode; data curation, L. Glänzer, L. Göpfert and I. Slabu; writing – original draft preparation, L. Glänzer; writing – review and editing, L. Glänzer, L. Göpfert, T. Schmitz-Rode and I. Slabu; visualization, L. Glänzer; supervision, I. Slabu; project administration, I. Slabu; funding acquisition, I. Slabu. All authors have read and agreed to the published version of the manuscript.

## Data availability

The data supporting this article have been included as part of the ESI.†

## Conflicts of interest

There are no conflicts to declare.

## Supplementary Material

TB-012-D4TB02052A-s001
