# Supplementary material for: Navigating predictions at nanoscale: a comprehensive study of regression models in magnetic nanoparticle synthesis
Source: J Mater Chem B. 2024 Nov 4;12(48):12652–64. doi: 10.1039/d4tb02052a (PMC11563307; doi:10.1039/d4tb02052a)
Supplement: TB-012-D4TB02052A-s001 [file TB-012-D4TB02052A-s001.pdf]

Supplementary Information (SI) for Journal of Materials Chemistry B.
This journal is © The Royal Society of Chemistry 2024

ARTICLE

Navigating Predictions at Nanoscale: A Comprehensive Study of Regression Models in Magnetic Nanoparticle Synthesis

Lukas Glänzer,[a] Lennart Göpfert[a], Thomas Schmitz-Rode[a] and Ioana Slabu†[a]

Received 00th January 20xx,
Accepted 00th January 20xx

DOI: 10.1039/x0xx00000x

S1 Dataset

The data for this study was extracted from a previously published synthesis setup[1]. Section 2.2.1 groups the data by type and offers brief descriptions of the individual system parameters. The following table gives the raw data before any manipulation by the described software.

$FeSO_4$ -7H_2O	$FeCl_3$-6H_2O	Iron Ion Ratio	V-IronSalts	NH_3	pH	V-Base	Coating Type	V-Coating	T-IronSalts	T-Base	T-Coil	FR-IronSalts	FR-Base	FR-Coating	Coil length	Quenching Position	MNP Size
0.01	0.015	1.5	100	500	14	2	LA	0.84	RT	RT	RT	1.1667	5.8888	-	10000	-	68.9
0.01	0.015	1.5	100	500	14	2	CA	28.824	RT	RT	RT	1.1667	5.8888	-	10000	-	15.8
0.01	0.015	1.5	250	500	14	2	LA	0.844	RT	RT	RT	2.222	4.888	-	10000	-	32.5
0.01	0.015	1.5	250	500	14	2	CA	28.824	RT	RT	RT	2.222	4.888	-	10000	-	15.7
0.01	0.015	1.5	250	500	14	2	CA	28.821	RT	RT	RT	2.35	4.667	3.444	10000	10000	18.1
0.01	0.015	1.5	250	500	14	2	CA	28.82	RT	RT	RT	2.4	4.644	3.25	10000	5000	14.9

1. *Institute of Applied Medical Engineering, Helmholtz Institute, Medical Faculty, RWTH Aachen University, Germany Address here.*
† Corresponding author: slabu@me.rwth-aachen.de; Tel.: +49 241 8089102
Supplementary Information available: [details of any supplementary information available should be included here]. See DOI: 10.1039/x0xx00000x

Please do not adjust margins

0.01	0.015	1.5	250	500	14	2	CA	28.822	RT	RT	RT	2.333	4.78	3.411	10000	35	4.3
0.01	0.015	1.5	250	500	14	2	CA	28.824	RT	RT	RT	2.4	4.644	3.444	10000	5000	-
0.01	0.015	1.5	250	500	14	2	CA	28.824	RT	RT	RT	2.4	4.644	3.444	10000	5000	24.2
0.01	0.015	1.5	250	500	14	2	CA	28.824	RT	RT	RT	2.4	4.644	3.444	10000	5000	19.6
0.01	0.015	1.5	250	500	14	2	CA	28.824	RT	RT	RT	2.4	4.644	3.444	10000	5000	21
0.01	0.015	1.5	250	500	14	2	CA	28.824	RT	RT	RT	2.4	4.644	3.444	10000	5000	14.3
0.01	0.015	1.5	250	500	14	2	CA	28.824	RT	RT	RT	2.4	4.644	3.444	10000	5000	19.7
0.01	0.015	1.5	250	500	14	2	CA	28.821	RT	RT	RT	2.4	4.644	3.444	10000	5000	-
0.01	0.015	1.5	250	500	14	2	CA	28.821	RT	RT	RT	2.4	4.644	3.444	10000	5000	16.5
0.01	0.015	1.5	250	500	14	2	CA	28.821	RT	RT	RT	2.4	4.644	3.444	10000	5000	14.7
0.01	0.015	1.5	250	500	14	2	CA	28.821	RT	RT	RT	2.4	4.644	3.444	10000	5000	13.1
0.01	0.015	1.5	250	500	14	2	CA	28.821	RT	RT	RT	2.4	4.644	3.444	10000	5000	12.4
0.01	0.015	1.5	250	500	14	2	CA	28.821	RT	RT	RT	2.4	4.644	3.444	10000	5000	11.4
0.01	0.015	1.5	250	500	14	2	CA	28.821	RT	RT	RT	2.4	4.644	3.444	10000	5000	9.3
0.04	0.06	1.5	250	500	14	2	CA	28.821	RT	RT	RT	2.4	4.644	3.444	10000	5000	-
0.04	0.06	1.5	250	500	14	2	CA	28.821	RT	RT	RT	2.4	4.644	3.444	10000	5000	10.7
0.04	0.06	1.5	250	500	14	2	CA	28.821	RT	RT	RT	2.4	4.644	3.444	10000	5000	-
0.04	0.06	1.5	250	500	14	2	CA	28.821	RT	RT	RT	2.4	4.644	3.444	10000	5000	-
0.04	0.06	1.5	250	500	14	2	CA	28.821	RT	RT	RT	2.4	4.644	3.444	10000	5000	10.8
0.04	0.06	1.5	250	500	14	2	CA	28.821	RT	RT	RT	2.4	4.644	3.444	10000	5000	-
0.04	0.06	1.5	250	500	14	2	CA	28.821	RT	RT	RT	2.4	4.644	3.444	10000	5000	-
0.01	0.015	1.5	250	500	14	2	Na-CA	44.118	RT	RT	RT	2.4	4.644	3.444	10000	5000	-
0.01	0.015	1.5	250	500	14	2	Na-CA	44.118	RT	RT	RT	2.4	4.644	3.444	10000	5000	-
0.01	0.015	1.5	250	500	14	2	Na-CA	44.118	RT	RT	RT	2.4	4.644	3.444	10000	5000	49.8
0.01	0.015	1.5	250	500	14	2	Na-CA	44.118	RT	RT	RT	2.4	4.644	3.444	10000	5000	-
0.01	0.015	1.5	250	500	14	2	Na-CA	44.118	RT	RT	RT	2.4	4.644	3.444	10000	5000	-
0.01	0.015	1.5	250	500	14	2	Na-CA	44.118	RT	RT	RT	2.4	4.644	3.444	10000	5000	14.6
0.01	0.015	1.5	250	500	14	2	Na-CA	44.118	RT	RT	RT	2.4	4.644	3.444	10000	5000	-
0.01	0.015	1.5	250	500	14	2	Na-CA	44.118	RT	RT	RT	2.4	4.644	3.444	10000	5000	-
0.01	0.015	1.5	250	500	14	2	CA	28.821	RT	RT	RT	2.3241	4.8056	3.4999	10000	2500	-
0.01	0.015	1.5	250	500	14	2	CA	28.821	RT	RT	RT	2.3241	4.8056	3.4999	10000	2500	-
0.01	0.015	1.5	250	500	14	2	CA	28.821	RT	RT	RT	2.3241	4.8056	3.4999	10000	2500	13.5
0.01	0.015	1.5	250	500	14	2	CA	28.821	RT	RT	RT	2.3241	4.8056	3.4999	10000	2500	-

This journal is © The Royal Society of Chemistry 20xx

Please do not adjust margins

Please do not adjust margins

0.01	0.015	1.5	250	500	14	2	CA	10.664	RT	RT	RT	2.4	4.644	3.25	10000	5000	-
0.01	0.015	1.5	250	500	14	2	CA	10.664	RT	RT	RT	2.4	4.644	3.25	10000	5000	14.5
0.01	0.015	1.5	250	500	14	2	CA	10.664	RT	RT	RT	2.4	4.644	3.25	10000	5000	12.9
0.01	0.015	1.5	250	500	14	2	CA	10.664	RT	RT	RT	2.4	4.644	3.25	10000	5000	12.2
0.01	0.015	1.5	250	500	14	2	CA	10.664	RT	RT	RT	2.4	4.644	3.25	10000	5000	10.351
0.01	0.015	1.5	250	500	14	2	CA	10.664	RT	RT	RT	2.4	4.644	3.25	10000	5000	8.7
0.01	0.015	1.5	250	500	14	2	CA	10.664	RT	RT	RT	2.4	4.644	3.25	10000	5000	8.8
0.01	0.015	1.5	250	500	14	2	CA	10.664	RT	RT	RT	2.4	4.644	3.25	10000	5000	14.3
0.01	0.015	1.5	250	500	14	2	CA	1.065	RT	RT	RT	2.4	4.644	3.25	10000	5000	-
0.01	0.015	1.5	250	500	14	2	CA	1.065	RT	RT	RT	2.4	4.644	3.25	10000	5000	13.5
0.01	0.015	1.5	250	500	14	2	CA	1.065	RT	RT	RT	2.4	4.644	3.25	10000	5000	17.5
0.01	0.015	1.5	250	500	14	2	CA	1.065	RT	RT	RT	2.4	4.644	3.25	10000	5000	15.9
0.01	0.015	1.5	250	500	14	2	CA	1.065	RT	RT	RT	2.4	4.644	3.25	10000	5000	15.1
0.01	0.015	1.5	250	500	14	2	CA	1.065	RT	RT	RT	2.4	4.644	3.25	10000	5000	14.2
0.01	0.015	1.5	250	500	14	2	CA	1.065	RT	RT	RT	2.4	4.644	3.25	10000	5000	13.7
0.01	0.015	1.5	250	500	14	2	CA	1.065	RT	RT	RT	2.4	4.644	3.25	10000	5000	36
0.01	0.015	1.5	250	500	14	2	CA	28.819	RT	RT	RT	2.3148	4.74	3.407	10000	10000	-
0.01	0.015	1.5	250	500	14	2	CA	28.819	RT	RT	RT	2.3148	4.74	3.407	10000	10000	-
0.01	0.015	1.5	250	500	14	2	CA	28.819	RT	RT	RT	2.3148	4.74	3.407	10000	10000	14.8
0.01	0.015	1.5	250	500	14	2	CA	28.819	RT	RT	RT	2.3148	4.74	3.407	10000	10000	-
0.01	0.015	1.5	250	500	14	2	CA	28.818	RT	RT	RT	2.333	4.722	3.574	10000	5000	-
0.01	0.015	1.5	250	500	14	2	CA	28.818	RT	RT	RT	2.333	4.722	3.574	10000	5000	-
0.01	0.015	1.5	250	500	14	2	CA	28.818	RT	RT	RT	2.333	4.722	3.574	10000	5000	16.4
0.01	0.015	1.5	250	500	14	2	CA	28.818	RT	RT	RT	2.333	4.722	3.574	10000	5000	-
0.01	0.015	1.5	250	500	14	2	CA	28.824	RT	RT	RT	2.287	4.694	3.472	10000	2500	-
0.01	0.015	1.5	250	500	14	2	CA	28.824	RT	RT	RT	2.287	4.694	3.472	10000	2500	-
0.01	0.015	1.5	250	500	14	2	CA	28.824	RT	RT	RT	2.287	4.694	3.472	10000	2500	16.1
0.01	0.015	1.5	250	500	14	2	CA	28.824	RT	RT	RT	2.287	4.694	3.472	10000	2500	-
0.01	0.015	1.5	250	500	14	2	CA	28.821	RT	RT	RT	2.315	4.63	3.444	10000	70	-
0.01	0.015	1.5	250	500	14	2	CA	28.821	RT	RT	RT	2.315	4.63	3.444	10000	70	7
0.01	0.015	1.5	250	500	14	2	CA	28.821	RT	RT	RT	2.32	4.63	3.444	10000	70	-
0.01	0.015	1.5	250	500	14	2	CA	28.821	RT	RT	RT	2.315	4.63	3.444	10000	70	-
0.01	0.015	1.5	250	500	14	2	CA	28.19	RT	RT	RT	2.333	4.78	3.411	10000	70	4

This journal is © The Royal Society of Chemistry 20xx

Please do not adjust margins

0.01	0.015	1.5	250	500	14	2	TEOS	46.7	RT	RT	RT	2.333	4.722	3.574	10000	5000	-
0.01	0.015	1.5	250	500	14	2	TEOS	46.7	RT	RT	RT	2.333	4.722	3.574	10000	5000	-
0.01	0.015	1.5	250	500	14	2	CA	2.643	RT	RT	RT	2.4	4.644	3.25	10000	5000	-
0.01	0.015	1.5	250	500	14	2	CA	2.643	RT	RT	RT	2.4	4.644	3.25	10000	5000	-
0.01	0.015	1.5	250	500	14	2	CA	2.643	RT	RT	RT	2.4	4.644	3.25	10000	5000	17.3
0.01	0.015	1.5	250	500	14	2	CA	2.643	RT	RT	RT	2.4	4.644	3.25	10000	5000	10.8
0.01	0.015	1.5	250	500	14	2	CA	2.643	RT	RT	RT	2.4	4.644	3.25	10000	5000	10.4
0.01	0.015	1.5	250	500	14	2	CA	2.643	RT	RT	RT	2.4	4.644	3.25	10000	5000	-
0.01	0.015	1.5	250	500	14	2	CA	2.642	RT	RT	RT	2.4	4.644	3.25	10000	5000	-
0.01	0.015	1.5	250	500	14	2	CA	2.642	RT	RT	RT	2.4	4.644	3.25	10000	5000	-
0.01	0.015	1.5	250	500	14	2	CA	2.642	RT	RT	RT	2.4	4.644	3.25	10000	5000	10.8
0.01	0.015	1.5	250	500	14	2	CA	2.642	RT	RT	RT	2.4	4.644	3.25	10000	5000	10.8
0.01	0.015	1.5	250	500	14	2	CA	2.642	RT	RT	RT	2.4	4.644	3.25	10000	5000	10.8
0.01	0.015	1.5	250	500	14	2	CA	2.642	RT	RT	RT	2.4	4.644	3.25	10000	5000	10.8
0.01	0.015	1.5	250	500	14	2	CA	2.642	RT	RT	RT	2.4	4.644	3.25	10000	5000	10.8
0.01	0.015	1.5	250	500	14	2	CA	2.642	RT	RT	RT	2.4	4.644	3.25	10000	5000	10.8
0.01	0.015	1.5	250	500	14	2	CA	2.642	RT	RT	RT	2.4	4.644	3.25	10000	5000	10.9
0.01	0.015	1.5	250	500	14	2	CA	2.642	RT	RT	RT	2.4	4.644	3.25	10000	5000	10.9
0.01	0.015	1.5	250	500	14	2	CA	2.642	RT	RT	RT	2.4	4.644	3.25	10000	5000	10.9
0.01	0.015	1.5	250	500	14	2	CA	2.642	RT	RT	RT	2.4	4.644	3.25	10000	5000	10.9
0.01	0.015	1.5	250	500	14	2	CA	2.642	RT	RT	RT	2.4	4.644	3.25	10000	5000	10.9
0.01	0.015	1.5	250	500	14	2	CA	2.642	RT	RT	RT	2.4	4.644	3.25	10000	5000	10.9
0.01	0.015	1.5	250	500	14	2	CA	2.643	RT	RT	RT	2.4	4.644	3.25	10000	5000	-
0.01	0.015	1.5	250	500	14	2	CA	2.643	RT	RT	RT	2.4	4.644	3.25	10000	5000	10.5
0.01	0.015	1.5	250	500	14	2	CA	1.906	RT	RT	RT	0.817	2.347	1.939	10000	5000	34.97
0.01	0.015	1.5	250	500	14	2	CA	1.5	RT	RT	RT	2.01	4.902	3.696	10000	5000	21.425
0.01	0.015	1.5	250	500	14	2	CA	3	RT	RT	RT	2.033	4.065	3.472	10000	5000	16.64
0.01	0.015	1.5	250	500	14	2	CA	1	RT	RT	RT	1.969	3.567	3.307	10000	2500	21.95
0.01	0.015	1.5	250	500	14	2	CA	2.645	RT	RT	RT	1.982	4.425	3.628	10000	2500	19.28
0.01	0.015	1.5	250	500	14	2	CA	2.642	RT	RT	RT	0.922	1.445	2.041	10000	2500	28.70089
0.01	0.015	1.5	250	500	14	2	CA	1.075	RT	RT	RT	1.908	3.252	3.58	10000	70	20.655
0.01	0.015	1.5	250	500	14	2	CA	25.07	RT	RT	RT	2.049	3.254	3.082	10000	70	90.21
0.01	0.015	1.5	250	500	14	2	CA	1.07	RT	RT	RT	1.25	1.115	2.26	10000	70	6.214

This journal is © The Royal Society of Chemistry 20xx

0.01	0.015	1.5	250	500	14	2	CA	2.64	RT	RT	RT	2.016	2.258	3.315	10000	70	7.44
0.01	0.015	1.5	250	500	14	2	CA	2.64	RT	RT	RT	2.252	4.486	3.559	10000	10000	34.3304
0.01	0.015	1.5	250	500	14	2	CA	2.64	RT	RT	RT	1.366	2.35	2.448	10000	10000	17.0923
0.01	0.015	1.5	250	500	14	2	CA	1.07	RT	RT	RT	1.453	1.779	2.267	10000	10000	55.7068
0.01	0.015	1.5	250	500	14	2	CA	1.07	RT	RT	RT	1.936	4.587	3.394	10000	10000	50.53
0.01	0.015	1.5	250	500	14	2	CA	1.07	RT	RT	RT	2.429	5.952	3.893	10000	10000	58.4751
0.01	0.015	1.5	250	500	14	2	CA	1.07	RT	RT	RT	-	-	-	10000	2500	13.87
0.01	0.015	1.5	250	500	14	2	CA	1.07	RT	RT	RT	2.083	3.042	3.633	10000	5000	13.87

This journal is © The Royal Society of Chemistry 20xx

Please do not adjust margins

S2 PCA Plots

The following PCA plots show the prediction surfaces of each presented method and the predictions of the data set by each model as red spheres. These visualizations further show the different prediction accuracies of the models.

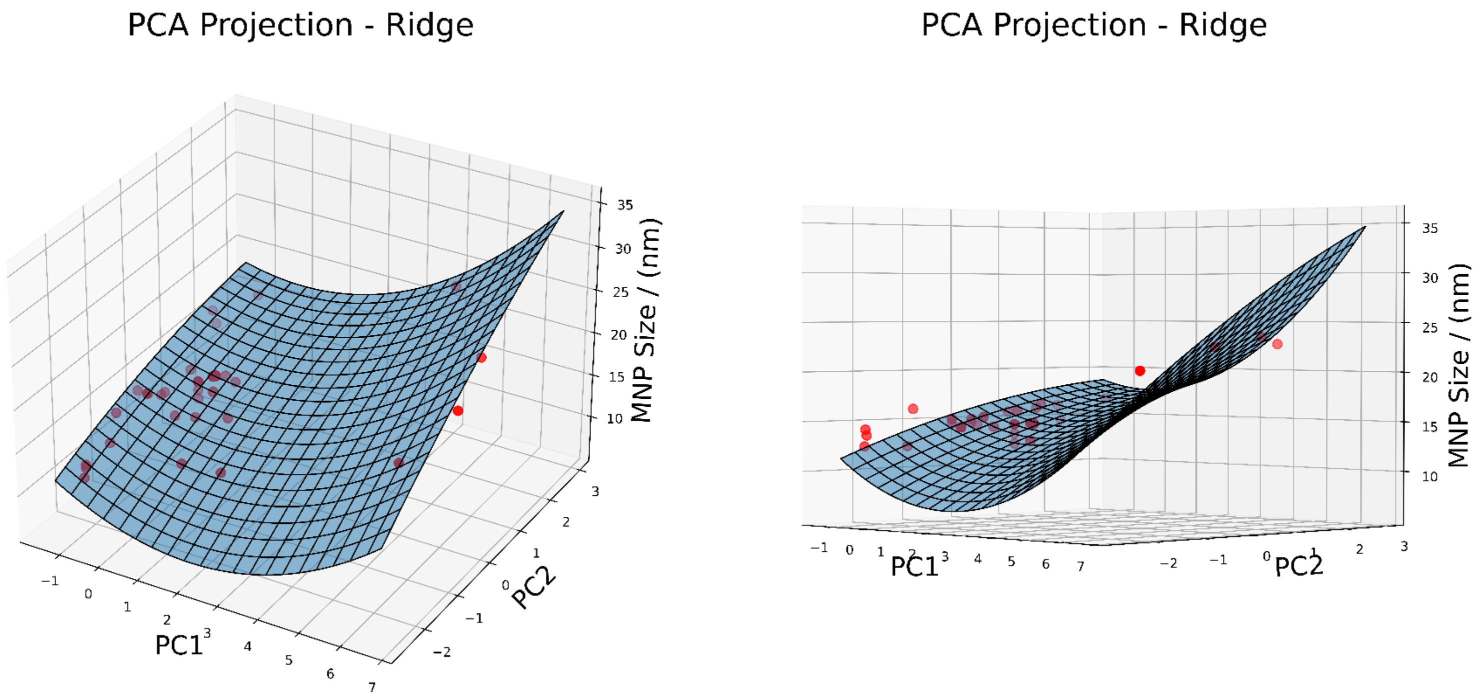

S2.A: PCA plot for Ridge Regression.

This journal is © The Royal Society of Chemistry 20xx

Please do not adjust margins

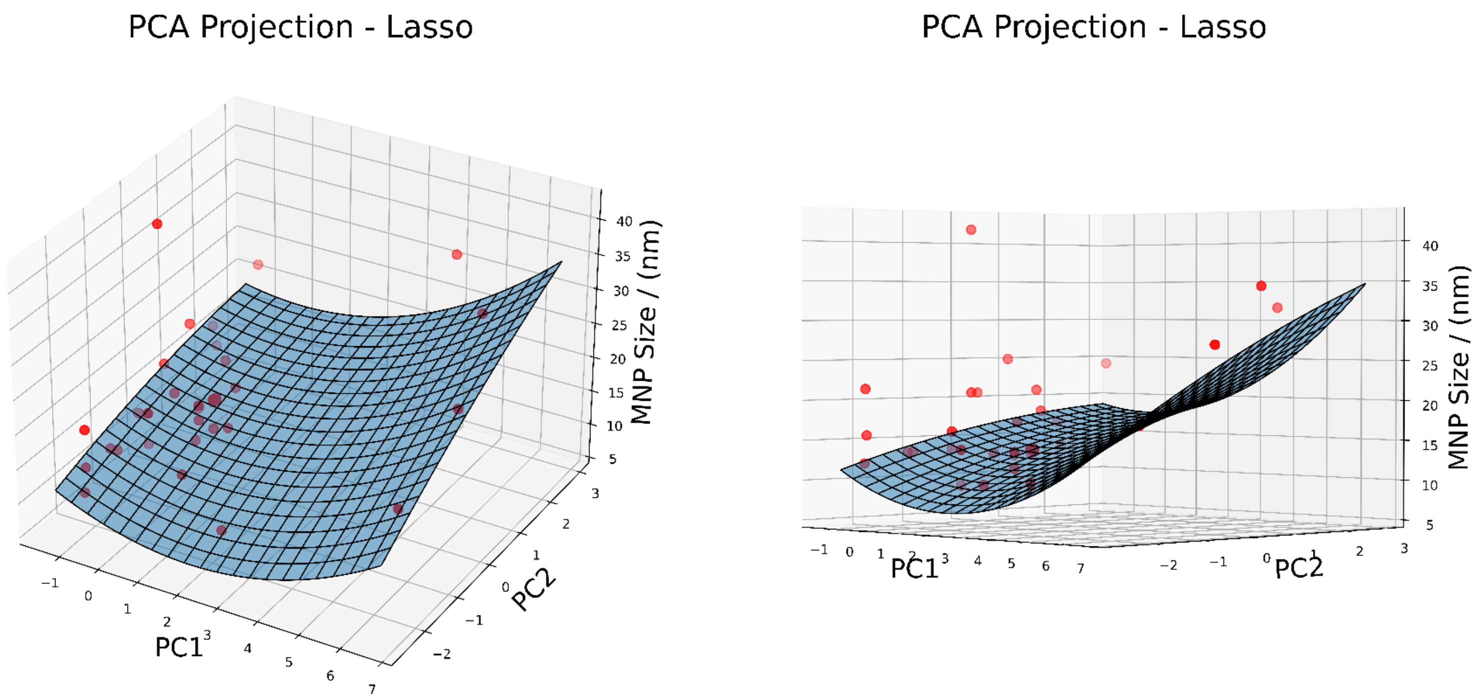

S2.B: PCA plot for Lasso Regression.

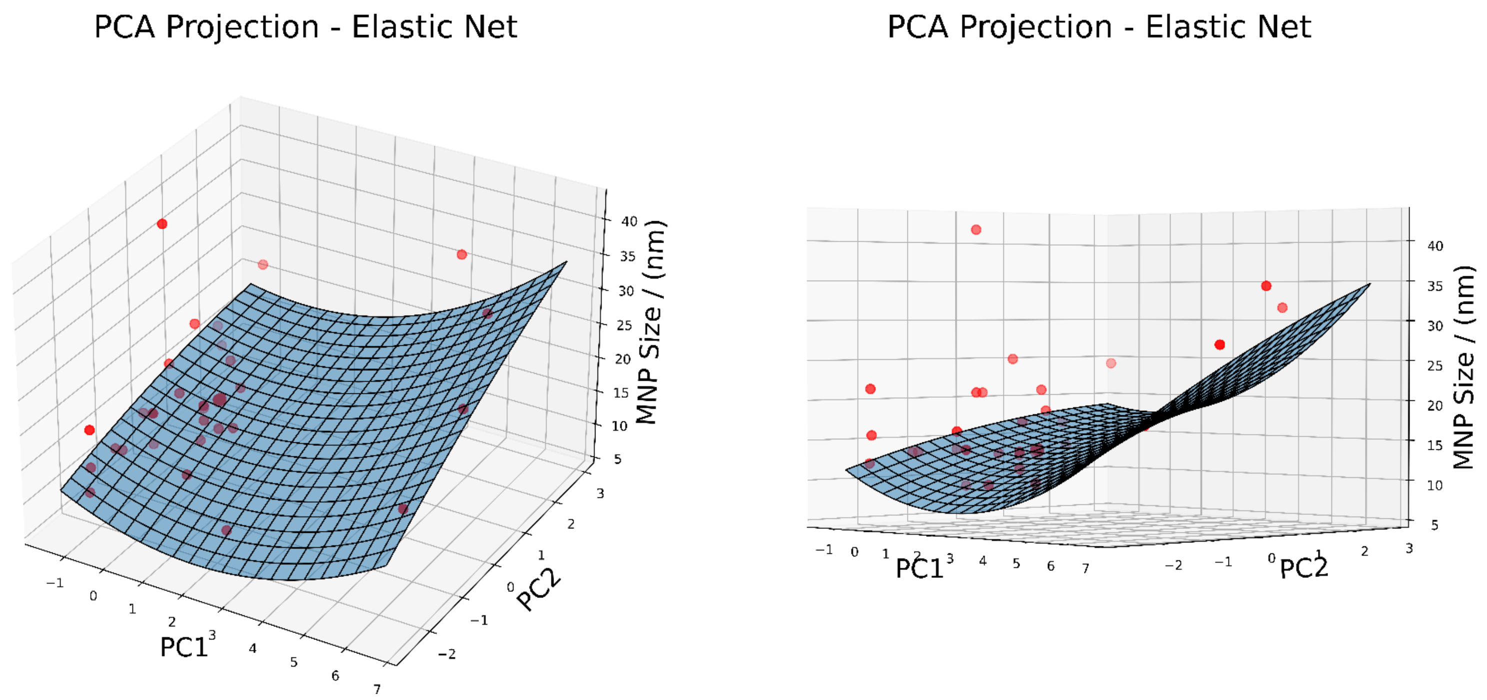

S2.C: PCA plot for Elastic Net Regression.

This journal is © The Royal Society of Chemistry 20xx

Please do not adjust margins

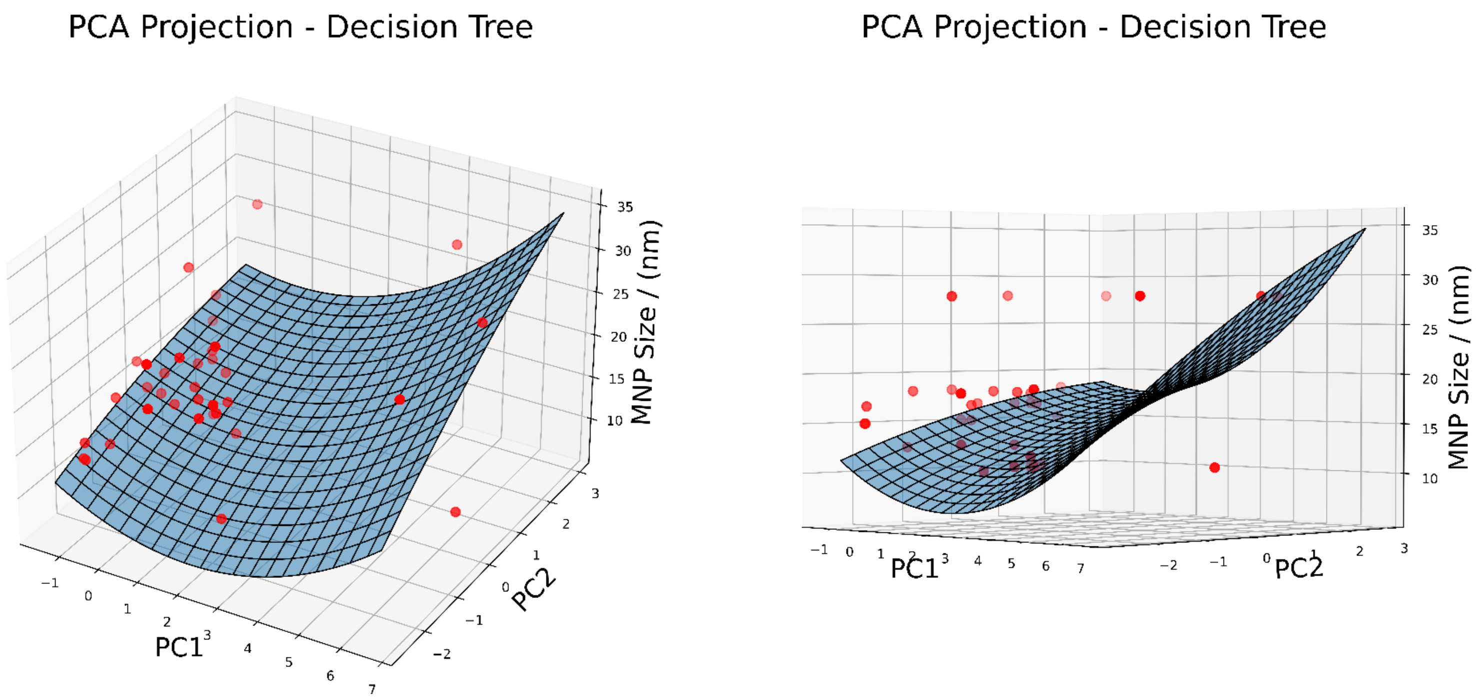

S2.D: PCA plot for Decision Tree Regression.

This journal is © The Royal Society of Chemistry 20xx

Please do not adjust margins

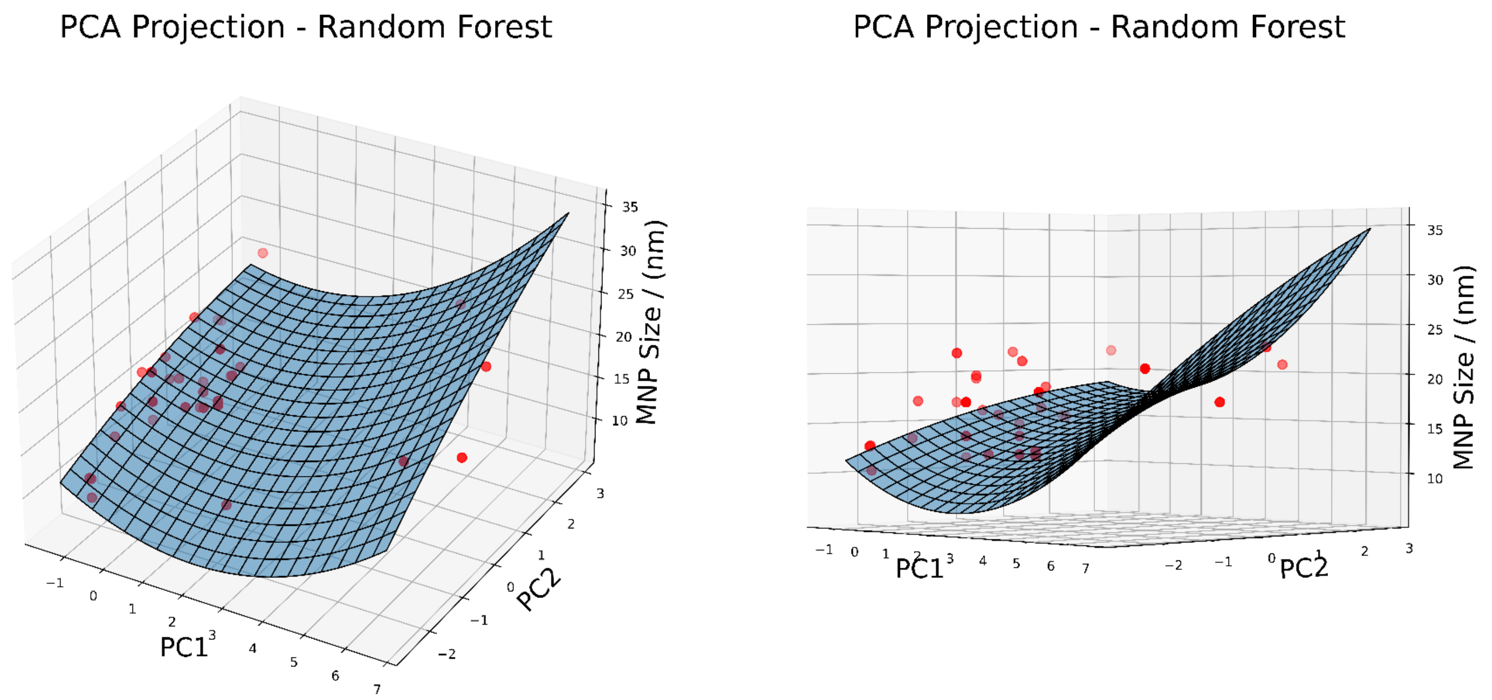

S2.E: PCA plot for Lasso Regression.

This journal is © The Royal Society of Chemistry 20xx

Please do not adjust margins

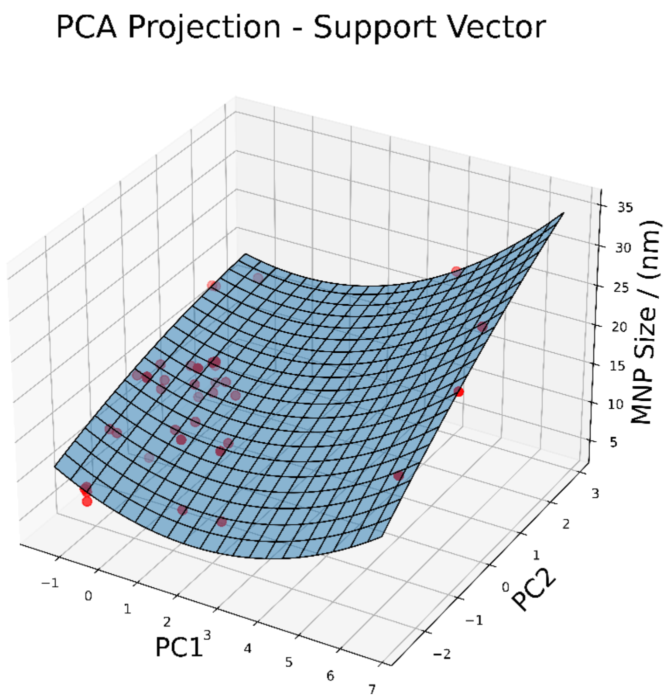

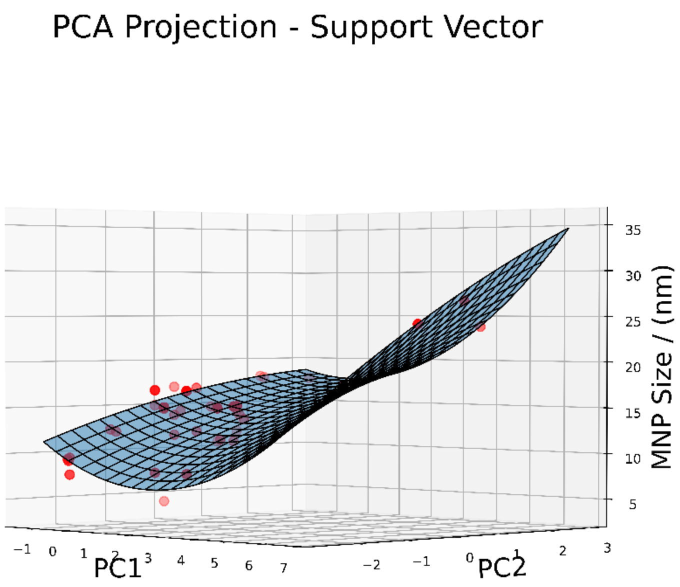

S2.F: PCA plot for Support Vector Regression.

Please do not adjust margins

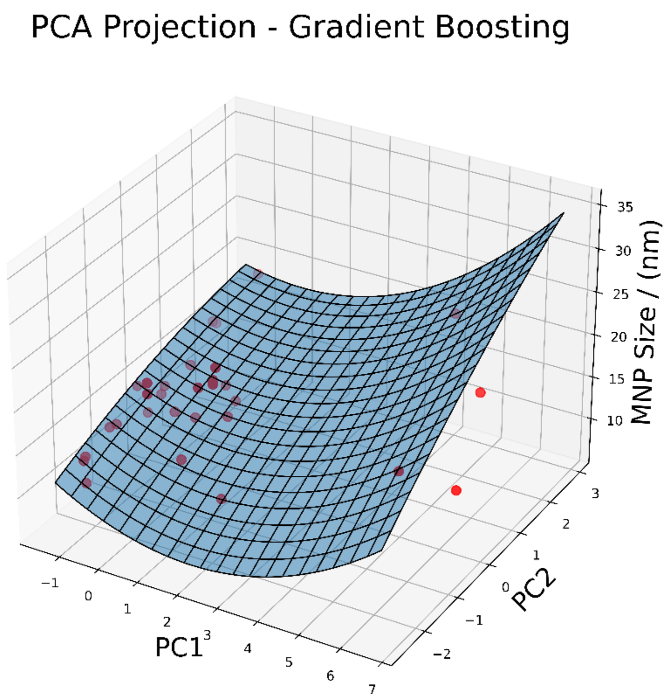

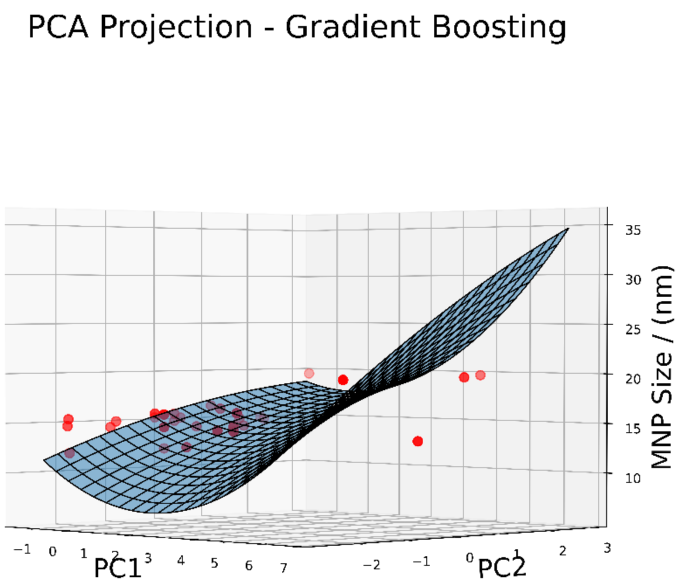

S2.G: PCA plot for Gradient Boosting Regression.

This journal is © The Royal Society of Chemistry 20xx

Please do not adjust margins

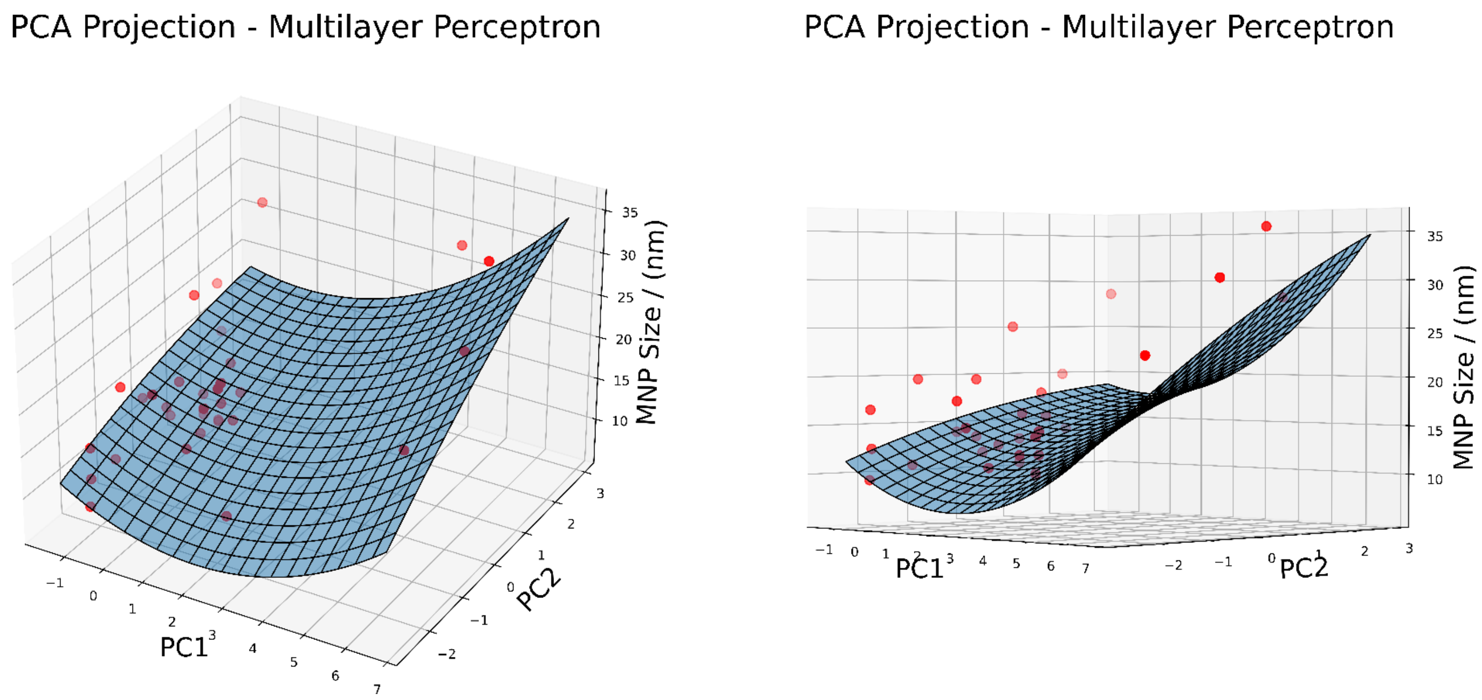

S2.H: PCA plot for Multilayer Perceptron Regression.

Notes and references

1 L. Göpfert, M. Schoenen, E. M. Buhl, T. Weirich, T. Schmitz-Rode and I. Slabu, A modularly built, scalable, and continuous millifluidic process enabling the synthesis of magnetic nanoparticles tailored for biomedical applications, *Chemical Engineering Journal*, 2024.

Please do not adjust margins
